# A Network of Chromatin Factors Is Regulating the Transition to Postembryonic Development in *Caenorhabditis elegans*

**DOI:** 10.1534/g3.116.037747

**Published:** 2016-12-22

**Authors:** Peter Erdelyi, Xing Wang, Marina Suleski, Chantal Wicky

**Affiliations:** Department of Biology, University of Fribourg, 1700 Fribourg, Switzerland

**Keywords:** genome-wide RNAi screen, chromatin, development, germline, P granules

## Abstract

Mi2 proteins are evolutionarily conserved, ATP-dependent chromatin remodelers of the CHD family that play key roles in stem cell differentiation and reprogramming. In *Caenorhabditis elegans*, the *let-418* gene encodes one of the two Mi2 homologs, which is part of at least two chromatin complexes, namely the Nucleosome Remodeling and histone Deacetylase (NuRD) complex and the MEC complex, and functions in larval development, vulval morphogenesis, lifespan regulation, and cell fate determination. To explore the mechanisms involved in the action of LET-418/Mi2, we performed a genome-wide RNA interference (RNAi) screen for suppressors of early larval arrest associated with *let-418* mutations. We identified 29 suppressor genes, of which 24 encode chromatin regulators, mostly orthologs of proteins present in transcriptional activator complexes. The remaining five genes vary broadly in their predicted functions. All suppressor genes could suppress multiple aspects of the *let-418* phenotype, including developmental arrest and ectopic expression of germline genes in the soma. Analysis of available transcriptomic data and quantitative PCR revealed that LET-418 and the suppressors of early larval arrest are regulating common target genes. These suppressors might represent direct competitors of LET-418 complexes for chromatin regulation of crucial genes involved in the transition to postembryonic development.

The concerted action of transcription factor networks and epigenetic regulators is required to ensure proper development of a multicellular organism. Together, these factors tightly control the transcriptional activity of the genome to allow a cell or a group of cells to acquire a specific fate at a given time of development. The highly conserved, ATP-dependent epigenetic modifier Mi2 is part of an abundant multi-protein complex in mammalian cells called NuRD (nucleosome remodeling and deacetylase). The first evidence for a developmental function of NuRD came from studies in mouse embryos, where a lack of the NuRD component Mbd3 compromised the differentiation potential of ES cells (Kaji *et al.* 2006, 2007). Molecular studies in ESCs revealed that Mi2β, as well as other NuRD components, suppresses the expression of pluripotency genes to allow transcriptional heterogeneity and, finally, proper lineage commitment (Reynolds *et al.* 2012, 2013). In the mouse hematopoietic system and during skin development the central component of NURD, Mi2β, is required for stem cell homeostasis and lineage choice (Kashiwagi *et al.* 2007; Yoshida *et al.* 2008). The *Drosophila* dMi2, together with Polycomb group proteins and the Hunchback transcription factor, regulates the transcriptional activity of the HOX genes during embryonic patterning (Kehle *et al.* 1998; McDonel *et al.* 2009). In addition, in *Arabidopsis*, the Mi2 homolog *Pickle* represses embryonic traits in root meristem cells and is required for proper postembryonic development (Ogas *et al.* 1997, 1999; Eshed *et al.* 1999).

The genome of *Caenorhabditis elegans* encodes two Mi2 homologs, LET-418 and CHD-3. LET-418 is required for postembryonic development, repression of the germline expression program in somatic cells, proper patterning of the vulva, and lifespan regulation (Solari and Ahringer 2000; von Zelewsky *et al.* 2000; Unhavaithaya *et al.* 2002; De Vaux *et al.* 2013). Lack of *chd-3* activity causes no obvious defects; however, when both *let-418* and *chd-3* functions are impaired, worm embryos arrest their development at the twofold stage. Genetic and biochemical analysis has revealed that LET-418 and CHD-3 act as multi-protein complexes. As part of the NuRD complex, they regulate early development. LET-418, together with the krüppel-like protein MEP-1 and the histone deacetylase HDA-1, is also a member of the so-called MEC complex, which is required for postembryonic development and repression of the germline expression program in somatic tissues (Passannante *et al.* 2010). Furthermore, LET-418, together with the histone H3K4 demethylase SPR-5/LSD1, prevents somatic reprogramming of the germline stem cells (Käser-Pébernard *et al.* 2014). Although studies in various organisms have revealed important developmental functions of Mi2 proteins and NuRD, the regulatory networks to which they contribute are not understood.

Postembryonic development in *C. elegans* is dependent on nutrient availability (Baugh 2013). In the absence of food, freshly hatched L1 (first larval stage) larvae undergo a developmental arrest (diapause) that is dependent on DAF-16/FOXO (Baugh and Sternberg 2006; Fukuyama *et al.* 2006). In diapaused larvae, germ cells and blast cells do not divide. It was shown recently that DAF-16/FOXO cell-nonautonomously controls the activity of both the *dbl-1*/TGFb and the *daf-12*/NHR signaling pathways, which both promote postembryonic development (Kaplan *et al.* 2015).

In our study, we show that LET-418 is required for the transition to postembryonic development. *let-418* mutants stop their development at the L1 stage and show no divisions of the germ cells and the blast cells. *let-418* L1 larvae look superficially similar to L1 diapaused larvae, but differ in their survival rate. A genome-wide RNAi screen identified 29 suppressors of *let-418* larval arrest, the majority of which encoded chromatin factors. All of the suppressors, except the histone methylase-encoding gene, *set-26*, suppressed both the developmental defect and the ectopic expression of germline genes associated with the developmental arrest of the *let-418* mutant. Finally, we show that a subset of the suppressors, together with LET-418, regulate common target genes, including germline genes and DAF-16 targets. We propose that an epigenetic network is controlling the transition to postembryonic development by acting on common target genes.

## Materials and Methods

### Culture conditions and C. elegans strains

The following strains were used in this study: wild-type (var. Bristol), *let-418*(*n3536ts*) (FR843), *ayIs6*[pBH47.70(hlh-8p::gfp) + pMH86(*dpy-20*(+))] (PD4666), *unc-119*(*e2498*::Tc1); *wIs51*[scmp::gfp + unc-119p::*unc-119*(+)] (JR667), *bnIs1*[pie-1p::gfp::*pgl-1* + unc-119p::*unc-119*(+)] (SS747), *bnIs1*[pie-1p::gfp::*pgl-1* + unc-119p::*unc-119*(+)];*let-418*(*n3536ts*) (FR1195), *set-26*(*tm2467*) (FR1496), *set-9*(*n4949*) (MT16426), *set-26*(*tm2467*);*let-418*(*n3536ts*) (FR1499), *set-9*(*n4949*);*let-418*(*n3536ts*) (FR1500), *set-26*(*tm2467*); *set-9*(*n4949*);*let-418*(*n3536ts*) (FR1506), *nurf-1*(*n4295*) (MT13649), and *nurf-1*(*n4295*);*let-418*(*n3536ts*) (FR1495). All *C. elegans* strains were maintained at 20° using standard conditions (Brenner 1974).

### Microscopy

Microscopy analyses were performed using a Zeiss axioplan 2 microscope. For brightfield pictures a DIC filter was used, and for fluorescence images the appropriate fluorescence filter was used. A Zeiss AxioCam color camera driven by AxioVision v4.8.2 software was applied for image acquisition.

### Starvation assay

Synchronized L4 stage animals were transferred to 25°. After 24 hr, the F1 generation of synchronized embryos was collected and equal amount of embryos were transferred to 20 ml of bacterium-free and OP50-containing S Medium. During the test, animals were shaken slowly at 25°. After every 24 hr, 100 μl of each sample were transferred to three OP50-seeded NGM plates; the survival rate was first determined and then the animals were kept at 15° to recover. Recovery rates were checked after 5–14 d.

### RNAi treatment

A previously described feeding technique was applied for RNAi treatment during the study (Timmons and Fire 1998). For the screen and for later experiments, RNAi clones from the Ahringer library were used (Kamath and Ahringer 2003). During the screen 12-well agar plates were used, whereas during other RNAi experiments 5 cm-wide petri dishes were applied. In all cases, HT115 bacteria containing empty L4440 vector were used as control.

### Genome-wide RNAi screen

For the genome-wide RNAi screen, RNAi treatment was performed as described above. Two L4 stage worms were added to each well containing different RNAi clones. The plates were then incubated for 8 d at 25°. After the first 24 hr period, the two P generation animals were removed. The F1 progeny was observed. Positive clones were retested in three independent experiments. The level of suppression was determined by comparison with a negative control (*let-418* worms on HT115 bacteria containing empty L4440 vector) and was shown to be statistically significant (*P* < 0.001, Fisher’s exact test, data not shown).

### P granule and blast cell screens

To determine the number of P granule-containing somatic cells, M cells, or V cells, 10 each of wild-type and **let-418*(n3536)* L4 stage animals carrying the appropriate transgene were transferred to RNAi plates and kept at 25°. Adults were removed after 24 hr of incubation and 40 F1 progeny were analyzed after 48 hr to determine the number of fluorescent cells. The Mann–Whitney *U*-test was used to determine the significance level.

### RNA isolation

Synchronized L4 stage animals were treated with RNAi at 25° as described above. After 24 hr, next generation embryos were collected in M9 solution from the gravid adults by hypochlorite treatment and were kept at 25° for 10 hr. Synchronized L1 worms were then put back onto the relevant RNAi plates for 3 hr at 25°. After incubation, larvae were harvested and RNA was isolated using a QIAGEN RNeasy Mini kit, according to the manufacturer’s protocol, combined with Precellys 24 0.5 mm glass beads to break open the animals.

### Quantitative real-time PCR (qRT-PCR)

cDNA was synthesized from total RNA using the QuantiTech Reverse Transcription Kit (QIAGEN). SensiFast SYBR No-ROX Kit (Bioline) was used for qPCR with a Corbett Rotor-Gene 6000 machine driven by Rotor-Gene 6000 v1.7 software. The primers were designed using Primer3 online software (http://bioinfo.ut.ee/primer3-0.4.0/primer3/). At least one primer of each primer pair aligned to an exon–exon junction. Primers designed for *ama-1* and *act-1* genes were used for normalization. The efficiency of the primer pairs was tested by setting a standard curve using a serial dilution of cDNA, and the specificity of the primers was monitored by analyzing the melting curves of each reaction. Data from triplicate reactions were analyzed using a 2^−ΔΔCt^ method. Biological replicates were used to confirm the results. For statistical analysis, we used the Student’s *t*-test to compare the relative mRNA level of different genetic variants.

### ModEncode analysis

Interpreted ChIP-seq data for each gene were downloaded from the modENCODE *C. elegans* online database (http://www.modencode.org) (Celniker *et al.* 2009). The list of affected genes was extracted using PAVIS peak annotation online software (http://manticore.niehs.nih.gov/pavis2/). Gene ontology (GO) analyses were performed using the DAVID bioinformatics database (Huang *et al.* 2009a,b). *P*-values were determined using Fisher’s exact test.

### Data availability

Further analysis of *daf-16/let-418* interaction is provided in Supplemental Material, Figure S1. Suppression of *let-418* developmental arrest by *set-26* is described in Figure S2. Suppression quantification of *let-418*-associated ectopic P granule expression, and M cell and V cell mitotic arrest, are provided in Figure S3, Figure S4, and Figure S5, respectively. LET-418 and DAF-16 common target genes are presented in Figure S6. Figure S7 shows the expression levels of selected DAF-16 targets measured in wild-type, *let-418*, *daf-16*, and *daf-16*;*let-418* mutant backgrounds.

## Results

### LET-418 promotes postembryonic development

**Figure 1:**
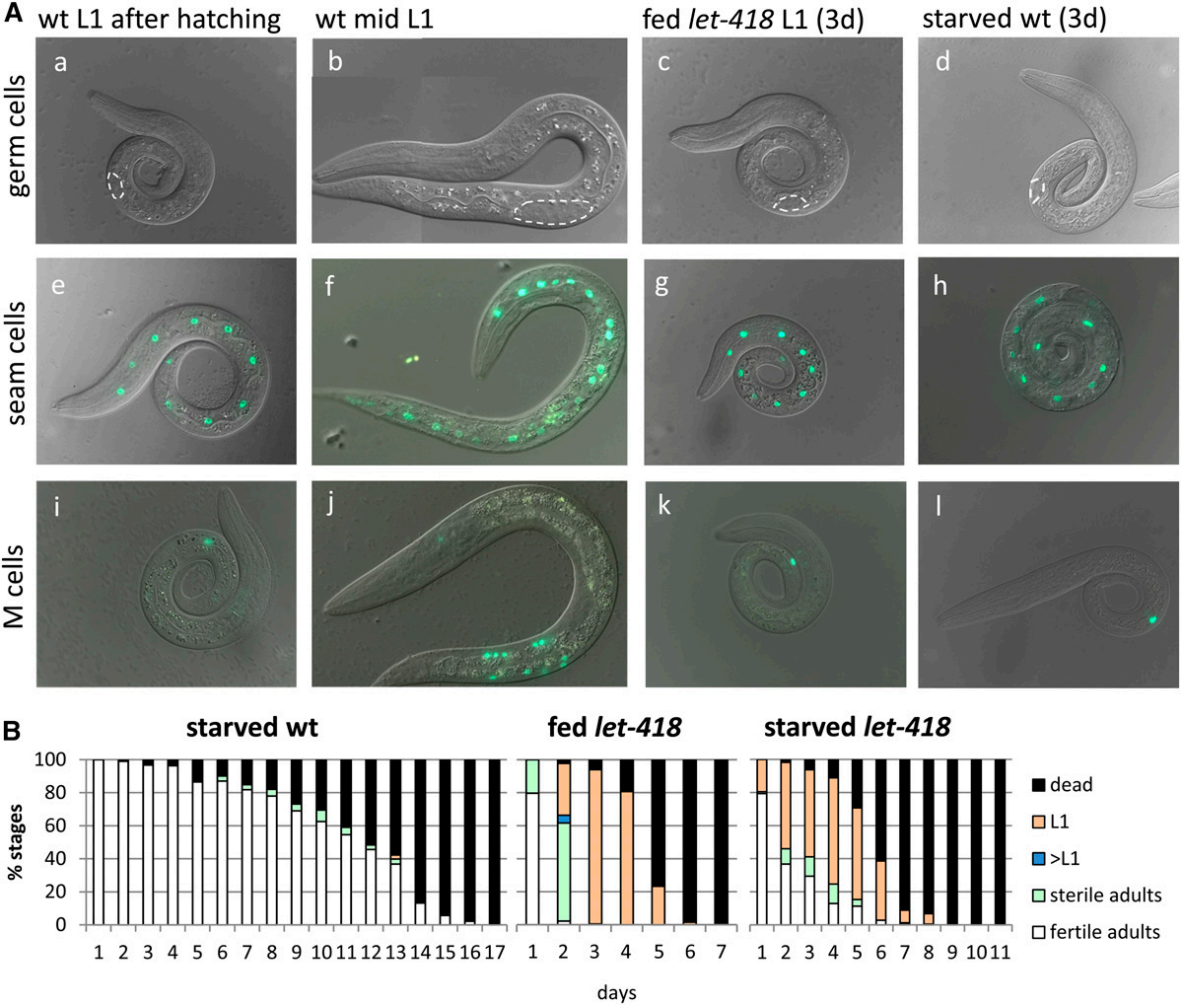
*let-418* mutants arrest as L1. (A) Primordial germ cells and blast cells are mitotically arrested in *let-418* mutants. a–d show differential interference contrast microscopy images of wt (a, b, and d) and *let-418* (c) developing larvae. e–h show V lineage and division pattern in wt (e, f, and h) and *let-418* (g) revealed by the reporter construct *scm*::*gfp*. (3d = days). i–l display mesoblast (M) lineage and division pattern in wt (i, j, and l) and *let-418* (k) larvae visualized by the reporter construct *hlh-8*::*gfp*. (B) LET-418 is required to survive and recover from starvation. Recovery and survival assays of indicated genotypes. Larvae were allowed to hatch at 25° in the presence or absence of food; starved larvae were returned to food following the indicated number of days. All larvae were maintained at 15°. wt, wild-type.

The progeny of temperature-sensitive *let-418(n3536)* mutants, grown at the restrictive temperature of 25°, fail to develop past the first larval (L1) stage. Examination of the mutant phenotype revealed that the two primordial germ cells (PGCs) Z2/Z3 do not divide in *let-418* L1 larvae (Figure 1A, c), whereas proliferation starts after hatching in wild-type animals (Figure 1A, a and b). In freshly hatched wild-type larvae, other cells, called blast cells, will also start to divide and differentiate to generate adult structures. To determine if these blast cells were also mitotically arrested in *let-418* mutants, we examined the V (seam) and the M (mesoblast) cell lineages.

Freshly hatched wild-type larvae possess 10 pairs of seam cells. All of them, except the most anterior ones (H0), are blast cells that will divide during development and give rise to different cell types, such as hypodermal cells, neurons, and glial cells (Sulston and Horvitz 1977) (Figure 1A, e and f). We analyzed the developmental pattern of the seam cells using the reporter gene *scm*::*gfp* (Terns *et al.* 1997) and found that, in *let-418* L1 larvae, the 10 pairs of V cells do not divide (Figure 1A, g). Furthermore, freshly hatched wild-type L1 larvae have a single M cell which, during further development, will give rise to two coelomocytes, 14 body wall muscle cells, and two sex myoblast cells (Figure 1A, i and j) (Sulston and Horvitz 1977). Using the *hlh-8*::*gfp* marker, which is expressed in all undifferentiated cells of the M lineage (Harfe *et al.* 1998), we observed a mitotic arrest of the M cell in *let-418* mutants (Figure 1A, k). In summary, our data suggest that, in arrested *let-418* L1 larvae, blast cells remain mitotically arrested. This growth arrest is similar to the L1 diapause, a dormant state that freshly hatched wild-type L1 larvae enter if no food is encountered (Baugh and Sternberg 2006; Fukuyama *et al.* 2006), and where the Z, V, and M cells are also mitotically arrested (Figure 1A, d, h, and l). Blast cell quiescence in L1 diapaused larvae is dependent on the DAF-16/FOXO transcription factor (Baugh and Sternberg 2006). In the absence of *daf-16* activity, blast cells are able to divide in starved L1 larvae. However, In *let-418* mutants, blast cell quiescence seems not to depend on DAF-16, as M cell division in *daf-16*;*let-418* double mutant worms could not be detected (Figure S1A). However, after close examination of the M cell morphology in *let-418* single and *daf-16*;*let-418* double mutants, we observed that a large proportion of *let-418* mutant animals were losing the expression of the M cell marker *hlh-8*::*gfp* after 3 d of developmental arrest, whereas *daf-16*;*let-418* double mutants or starved wild-type animals did not (Figure S1, B and C). This observation suggested that an absence of *daf-16* does not suppress mitotic quiescence, but instead prevents the occurrence of some other defects in the *let-418* mutant larvae, as indicated by the loss of expression of the M cell marker (Figure S1, B and C).

In addition to blast cell quiescence, starved L1 larvae exhibit an extended survival rate in the absence of food and, when returned to food, can recover from this diapause state and resume their development. We measured the survival rate of arrested *let-418* L1 larvae. Both fed and starved *let-418* L1 mutants showed a significantly decreased survival rate as compared to starved wild-type L1 larvae (Figure 1B). However, starved *let-418* L1 larvae exhibited a better survival rate than fed *let-418* worms (Figure 1B), indicating that starvation increases the resistance of *let-418* mutants. We also tested if a lack of *daf-16* activity would improve the survival rate of *let-418* in fed conditions, but we could not observe any effect (Figure S1D). Absence of *daf-16* activity prevents the occurrence of some defects in the *let-418* larvae, but not the survival rate. Next, we determined the recovery rate of *let-418(ts)* mutants by returning them to the permissive temperature after different time points. Interestingly, starved *let-418(ts)* L1 larvae recovered better than fed *let-418(ts)* mutants, which almost completely lost their potential to resume development after 3 d at restrictive temperature (Figure 1B) and exhibited a *hlh-8*::*gfp* expression pattern comparable to starved wild-type L1 larvae (Figure S1, B and C). Obviously, starvation protects *let-418* worms and preserves their developmental potential. In summary, our data demonstrated that *let-418* L1 larvae stop postembryonic development in a process that looks superficially similar, but is not identical to, that observed in a starvation-induced L1 diapause.

### A genome-wide RNAi screen for suppressors of the let-418 L1 arrest phenotype mostly identified chromatin factors

To better understand the role of LET-418 during the transition to postembryonic development and uncover its regulatory network, we performed a genome-wide RNAi screen to identify suppressors of L1 arrest. Out of the 16,757 genes of the Ahringer library (Timmons *et al.* 2001; Kamath *et al.* 2003), 29 suppressors were identified (Table 1). Among the 29 suppressor genes, 24 are known to be involved in chromatin regulation. At least 15 of them encode proteins that belong to activating chromatin complexes, such as the ISWI/NURF, SWR1, NuA4, KAT8/MOF, and COMPASS complexes (Table 1).

**Table 1:** *let-418* suppressor identities

Complex	Gene Name	Human Ortholog	Description
ISWI/NURF	***isw-1****^a^*	SMARCA1, SMARCA5	ATPase component of a nucleosome remodeling factor (NURF)-like complex
	***nurf-1****^b^*	CECR2	Acts with *isw-1* in vulval development
SWR1	***C17E4.6****^b^*	VPS72	Gene expression regulator
	***zhit-1****^b^*	ZNHIT1	HIT-type zinc finger protein
	***arp-6****^b^*	ACTR6	Actin-related protein
	***htz-1****^c^*	H2AFV, H2AFZ	Regulate gene expression with SWR1 in pharynx
SWR1/NuA4	***gfl-1****^b^*	YEATS4	Transcription factor
	***mrg-1****^a^*	MRG15	Chromodomain-containing protein, promotes cell-proliferation
NuA4	***zk1127.3****^c^*	—	Unknown
	***mys-4****^b^*	MYST3	MYST family histone acetyltransferase
COMPASS	***cfp-1****^b^*	CXXC1	CFP1 (CpG-binding protein, CXXC Finger Protein 1) homolog
	***dpy-30****^b^*	DPY-30	Hermaphrodite dosage compensation and normal male development
	***wdr-5.1****^b^*	WDR5, WDR5B	WD40 repeat-containing proteins, regulate H3K4 methylation levels
	***hcf-1****^b^*	HCF-1	Regulates cell division and mitotic histone modification
	***set-2****^b^*	SETD1A, SETD1B	Histone H3K4 methyltransferase, germline development, postembryonic development
Polycomb	***mes-3****^b^*	—	Required maternally for normal germline development and for anteroposterior patterning
	***mes-6****^b^*	EED	Required maternally for normal germline development and for anteroposterior patterning
	***mes-2****^b^*	EZH2 isoform b	Required maternally for normal germline development and for anteroposterior patterning
KAT8/MOF-like	***sumv-1****^c^*	INO80D	Encodes a protein with similarity to the KAT8 NLS3 nonenzymatic subunit of the mammalian KAT8/MOF histone acetyltransferase complex
	***sumv-2****^c^*	—	Encodes a protein with similarity to the KAT8 NLS3 nonenzymatic subunit of the mammalian KAT8/MOF histone acetyltransferase complex
Other	***C06A5.3****^c^*	PSIP1, HDGFL1, HDGF	Involved in reproduction, PWWP domain-containing protein
	***mes-4****^a^*	ASH1L, EZH1, EZH2	SET domain-containing protein, required maternally for normal germline development
	***set-26****^c^*	KMT2E, SETD5	PHD-zinc finger and a SET domain
	***lex-1****^c^*	ATAD2, ATAD2B	Contains an ATPase domain and a bromodomain, positively regulate expression of repetitive sequences
	*lsl-1**^b^*	ZBTB20, ZBTB45, ZBTB7C, ZFP57, ZNF296	LSY-2-like
	*math-33**^c^*	USP-7	Encodes a protein with a meprin-associated Traf homology (MATH) domain
	*T19B4.5**^c^*	—	Unknown
	*gtbp-1**^b^*	G3BP1, G3BP2	Predicted to have nucleotide-binding activity and nucleic acid-binding activity
	*M03C11.3**^c^*	—	Unknown

Chromatin factors are shown in bold. SWR1, Swi2/Snf2-related ATPase 1; MYST, Moz, Ybf2/Sas3, Sas2, Tip60; KAT, lysine (K) acetyltransferase; NLS, nuclear localisation signal; MOF, males absent on the first; PHD, plant homeodomain; SET, Su(var)3-9, Enhancer-of-zeste and Trithorax; LSY, laterally symmetric; MATH, Meprin and TRAF-Homology.

a Strong suppression, larvae reach L4/adulthood.

b Weak suppression, larvae bypass L1 arrest.

c Middle suppression, larvae reach L2/L3 stage.

The proteins NURF-1 and ISW-1 are homologs of the ISWI complex members. Reduction of the functions of *isw-1* and *nurf-1* has already been shown to suppress the larval-lethal phenotype of *mep-1(q660)* and *let-418(n3536)* (Andersen *et al.* 2006). Our data confirmed these results.

The proteins encoded by *zhit-1*, *arp-6*, *C17E4.6*, *mrg-1*, and *gfl-1* are homologous to members of the mammalian SWR1 complex (Lu *et al.* 2009), which carries out the incorporation of histone variants such as H2A.Z (Mizuguchi *et al.* 2004). Depletion of members of the SWR1 complex or of HTZ-1, the *C. elegans* homolog of H2A.Z, suppresses the *let-418* phenotype. Moreover, MRG-1 and GFL-1 are shared members of the NuA4 acetylation complex, which also includes the products of the suppressor gene homologs ZK1127.3 and MYS-4. Both the SWR1 and NuA4 complexes have been shown to exhibit antisilencing activity through chromatin remodeling, histone variant deposition, and histone acetylation [reviewed in Lu *et al.* (2009)]. Genetic interaction of *let-418* with components of these two complexes suggests that LET-418 could prevent the establishment of active chromatin at various loci in the genome.

*hcf-1*, *dpy-30*, *wdr-5.1*, *cfp-1*, and *set-2* encode worm homologs of the COMPASS complex (Li and Kelly 2011; Shilatifard 2012). The COMPASS complex is responsible for H3K4 methylation, which correlates with transcriptional activity and might antagonize *let-418* function during development.

*mes-2/3/6* encode members of a worm PRC2 repressive complex, which is considered to be a regulator of pluripotency and differentiation in both mammals and *C. elegans* (Boyer *et al.* 2006; Lee *et al.* 2006; Yuzyuk *et al.* 2009). Absence of *let-418* activity could lead to mislocalization of Polycomb proteins and the repression of inappropriate genes, and the suppression effect obtained by deactivating PRC2 components may indicate the need to remove the repression of these genes to allow development in a *let-418* background (see also *Discussion*).

Among the RNAi suppressors, we found two genes, *set-26* and *set-9*, that showed 97% identity at the level of their nucleotide sequence. To test if both genes act as suppressors, we generated *set-26*;*let-418* and *set-9*;*let-418* double mutants as well as *set-26*;*set-9*;*let-418* triple mutants. We found that only the mutation in *set-26*, but not in *set-9*, could suppress the L1 arrest phenotype of *let-418* (Figure S2). Consistently, the rate of suppression in the *set-26*;*let-418* double mutants was identical to that of the triple mutants *set-26*;*set-9*;*let-418*, suggesting that only *set-26*, but not *set-9*, is a *let-418* suppressor (Table 1). The reason that we identified *set-9* as a suppressor was likely due to the fact that *set-9(RNAi)* cross-inhibited *set-26* expression. *set-26* encodes a H3K9 methyltransferase that is involved in the transgenerational sterility of *spr-5* mutants lacking H3K4 demethylase activity (Greer *et al.* 2014). Some interesting aspects of the *set-26* suppression effect will be further discussed below.

We also identified the two genes, *sumv-1* and *sumv-2*, whose protein products are members of a putative worm KAT8/MOF histone acetyltransferase complex (Rea *et al.* 2007). This chromatin-activating complex is known to play a role during vulval development (Yucel *et al.* 2014).

The autosome-specific H3K36 methyltransferase, MES-4, was also found among the suppressors (Furuhashi *et al.* 2010; Rechtsteiner *et al.* 2010). MES-4 regulates active chromatin and might be required for the transcription of genes that are induced in the absence of *let-418*.

*lex-1* encodes a bromodomain-containing ATPase, which was shown to promote gene expression in the context of repetitive sequences (Tseng *et al.* 2007). LEX-1 might also be involved in the expression of genes that are induced in the absence of *let-418*. *C06A5.3* encodes a protein with a PWWP domain that interacts with methylated histone H4 at lysine 20. This specific histone modification is known to recruit cell cycle checkpoint proteins (Wang *et al.* 2009). In the absence of *let-418* activity, the C06A5.3 protein might interact abnormally with checkpoint proteins that could block the blast cell cycle.

The remaining five genes are not known to be directly associated with chromatin function. *math-33* participates in protein metabolism. It was first identified as a cde (cosuppression defective) gene (Robert *et al.* 2005). More recently, it was shown to be part of the deubiquitylation machinery required for the establishment of embryonic polarity (McCloskey and Kemphues 2012). Absence of *math-33* activity could destabilize proteins responsible for the *let-418* phenotype. *lsl-1* encodes a zinc finger transcription factor whose function is unknown, and *gtbp-1* was shown to be associated with stress granules in human cells (Jedrusik-Bode *et al.* 2013). M03C11.3 and T19B4.5 encode gene products whose functions are not known yet.

In summary, we isolated mainly chromatin factors in our suppressor screen, suggesting that there is a need to modify the expression of a large number of target genes to suppress *let-418*-associated defects.

### Suppression of let-418-associated somatic P granules

**Figure 2:**
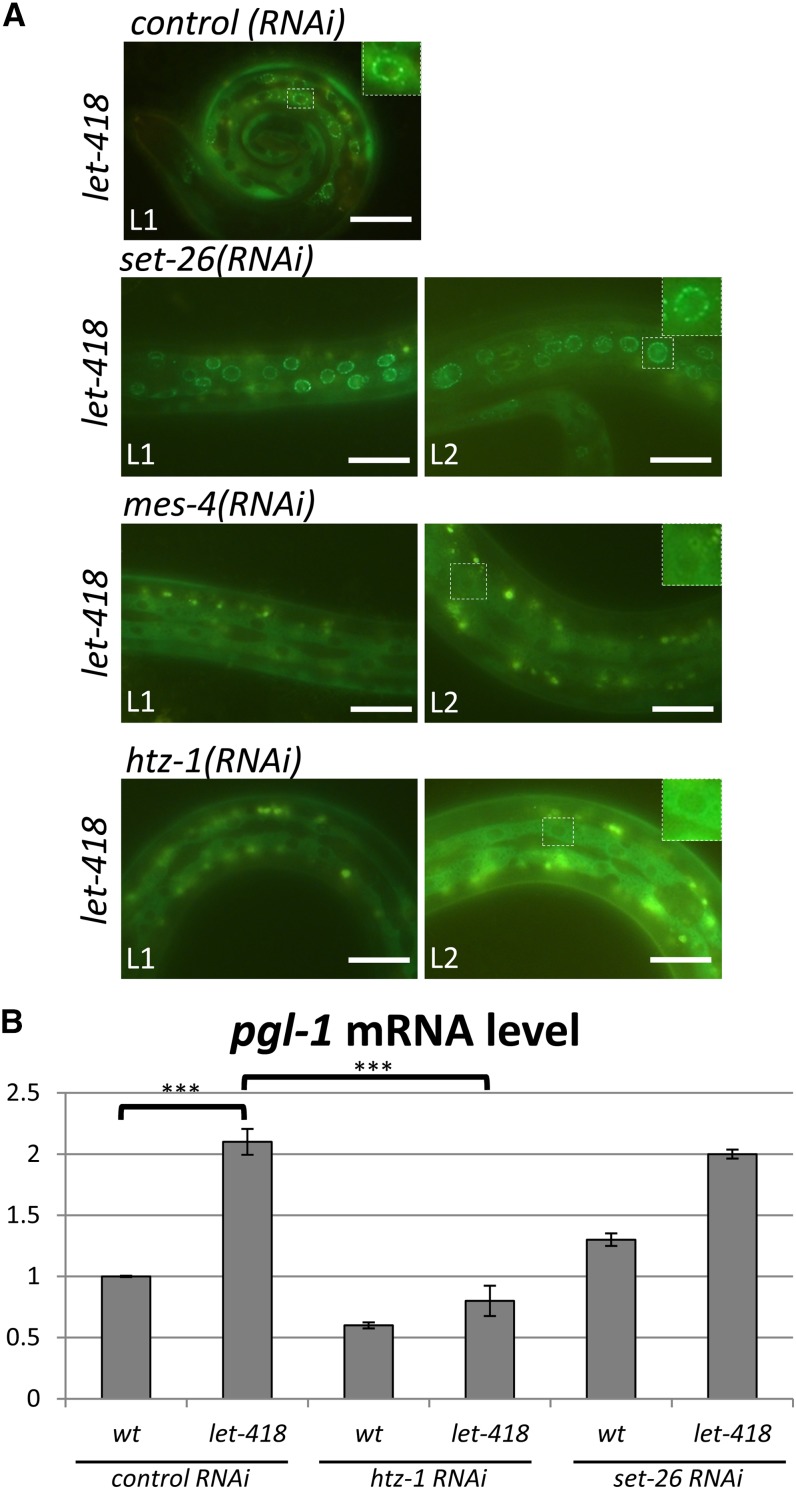
Suppression of ectopic P granule expression. (A) Perinuclear P granules are observed in somatic cells of *let-418* and *set-26(RNAi)* worms, but are missing in animals that are fed with *mes-4* or *htz-1* RNAi. Presence of the P granule was revealed by the reporter construct *pgl-1*::*gfp*. Bar, 20 µm. (B) *pgl-1* mRNA level is decreased in *htz-1*;*let-418* but not in *set-26*;*let-418* animals. mRNA level of *pgl-1* was measured by qRT-PCR and represented as fold induction of mRNA expression *vs.* wt. Total mRNA was isolated from wt and *let-418* L1 animals treated with the indicated suppressor RNAi. *** indicates a P value ≤ 0.001 mRNA, messenger RNA; qRT-PCR, quantitative real-time polymerase chain reaction; RNAi, RNA interference; wt, wild-type.

While P granules appear only in the PGCs Z2/Z3 of wild-type L1 animals, *let-418* mutants also show ectopic P granule components around somatic nuclei (Unhavaithaya *et al.* 2002). Using the reporter construct *pie-1p*::*gfp*::*pgl-1* to monitor the presence of P granules, *let-418* mutants show an average of 12 P granule-positive somatic cells (Figure S3). We tested if our suppressor RNAis were able to reduce the ectopic expression of the reporter construct in a *let-418* background. Of the 29 suppressors, 28 significantly decreased the number of cells showing ectopic P granules in *let-418* mutants (Figure 2A and Figure S3).

Interestingly, *let-418* animals fed on *set-26* RNAi grow to the L2/L3 stage, but a large number of P granule-containing cells are still detected (Figure 2A). In contrast, *let-418* animals treated with *htz-1* or *mes-4* RNAi develop at least to the same stage, if not further, but show no ectopic P granules (Figure 2A). To further analyze the expression of genes encoding P granule components in *let-418*;*set-26(RNAi)* animals, we measured the mRNA level of *pgl-1*. Consistent with the *pie-1p*::*gfp*::*pgl-1* reporter analysis, the *pgl-1* expression level was elevated in *let-418* L1 larvae, but we observed no reduction in *let-418*;*set-26* animals (Figure 2B). In contrast to most of the suppressors that restored development and restricted P granules to the germline, lack of *set-26* activity only suppressed developmental arrest. This result shows that the presence of ectopic P granules does not prevent the onset of postembryonic development, and suggests that the regulation of development and repression of germline gene expression in somatic cells could be controlled by distinct functions of LET-418. Another interpretation of this result could be that the absence of *set-26* activity sets up a sensitized background for ectopic expression of P granule components. Consistent with this hypothesis is the slight *pgl-1* overexpression observed in *set-26(RNAi)* (Figure 2B).

### Suppression of the let-418-associated mitotic arrest of the blast cells

We followed the division pattern of the M cell using *hlh-8*::*gfp*, which is expressed only in undifferentiated cells of the M cell lineage (Harfe *et al.* 1998). In L1 arrested *let-418(n3536)* animals, the M cell did not divide (Figure 1A, k). When *let-418* mutant animals were treated with the suppressor RNAis, we detected divisions of the M blast cell in all cases (Figure S4), although the level of M cell divisions did not fully correspond to the observed developmental stage of the worms. Thus, our data suggest that M cell division and larval growth are uncoupled in *let-418* animals. A delay in M cell division compared to the growth rate was previously reported in a *daf-2* mutant lacking insulin receptor activity (Chen and Baugh 2014).

**Figure 3:**
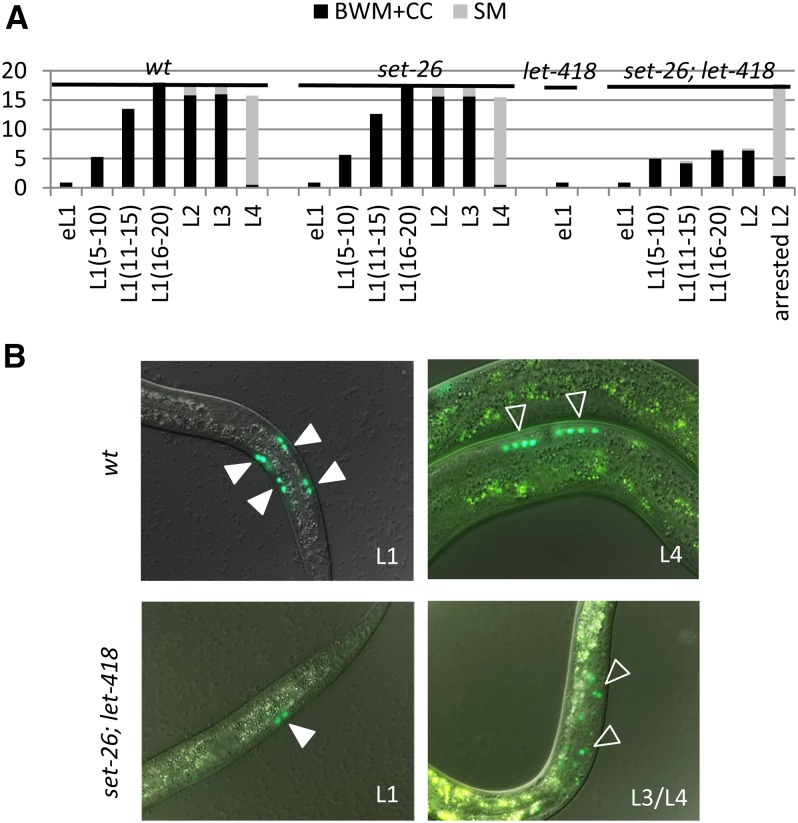
*set-26* suppression of M cell mitotic arrest. (A) Inactivation of *set-26* in *let-418* mutant background allows the M cell to divide. However, *set-26*;*let-418* animals show reduced numbers of muscle cell precursors compared to wt. Numbers in brackets correspond to the number of germ cells indicative of the developmental stage. (B) M cell division pattern is revealed by the reporter construct *hlh-8*::*gfp*. Developing L1 shows BWM cell precursors (filled arrowheads) and L4 larva show SM precursors (open arrowheads). SM precursor cells are mislocalized in *set-26*;*let-418* animals. BWM, body wall muscle; CC, coelomocytes; eL1, early L1 with four germ cells (Z1–4); SM, sex myoblast; wt, wild-type.

In *let-418* worms treated with *set-26* RNAi, we observed small persisting GFP-positive cells in the central body region, where sex myoblasts are dividing and differentiating (Figure 3, A and B). This observation suggests that *let-418*;*set-26(RNAi)* worms arrest their development at a stage where sex myoblasts are dividing but are not yet differentiating (L3 stage). In addition, these GFP-positive cells are disorganized, indicating defects in sex muscle patterning in *let-418*;*set-26* worms.

Freshly hatched larvae possess 10 pairs of seam cells. We monitored the developmental fate of the seam cell lineage using animals carrying a *scm*::*gfp* transgene (Terns *et al.* 1997). *let-418* L1 larvae exhibited no seam cell division (Figure 1A, g and Figure S5); However, inactivation of all 29 suppressor genes by RNAi resulted in seam cell divisions to different extents (Figure S5). Similar to our previous observations regarding the M cell divisions, the number of seam cells was lower than expected from the apparent developmental stage of the animal (Figure S5). Altogether, our results show that RNAi of the suppressors can restore blast cell division in a *let-418* mutant background to some extent, although none of the worms could reach adulthood.

### LET-418, NURF-1, and HTZ-1 are regulating common target genes

**Figure 4:**
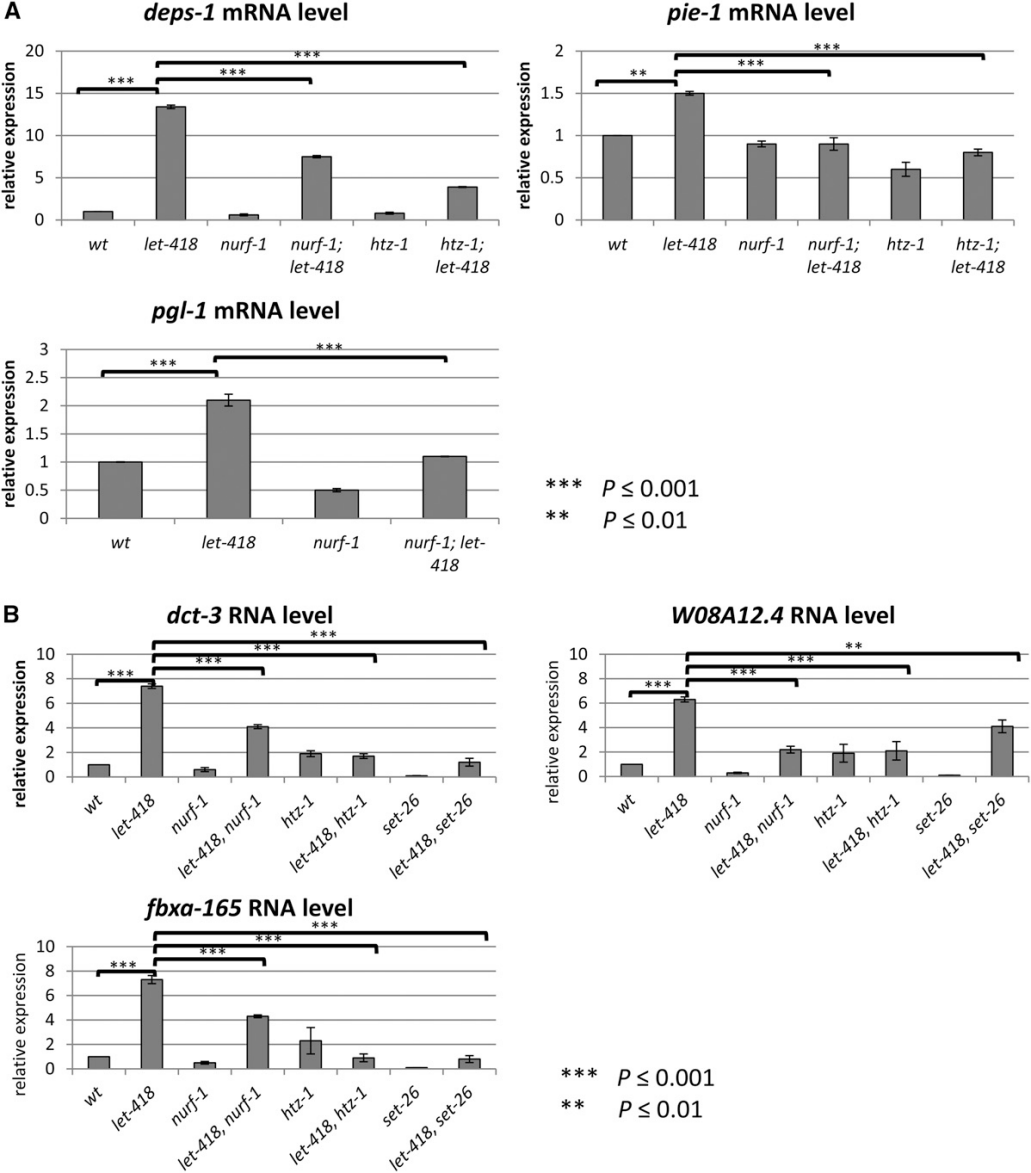
LET-418, NURF-1, and HTZ-1 regulate common target genes. (A) Upregulation of the germline gene expression in *let-418* depends on NURF-1 and HTZ-1. mRNA levels of *deps-1*, *pie-1*, and *pgl-1* were measured by qRT-PCR and represented as fold induction of mRNA expression *vs.* wt. Total mRNA was isolated from wt and *let-418* L1 animals treated with the indicated suppressor RNAi. *ama-1* was used to normalize. (B) Upregulation of DAF-16 target expression in *let-418* depends on NURF-1 and HTZ-1. mRNA levels of *dct-3*, *W08A12.4*, and *fbxa-165* were measured by qRT-PCR and represented as fold induction of mRNA expression *vs.* wt. Total mRNA was isolated from wt and *let-418* L1 animals treated with the indicated suppressor RNAi. *ama-1* was used to normalize. mRNA, messenger RNA; qRT-PCR, quantitative real-time polymerase chain reaction; RNAi, RNA interference; wt, wild-type.

**Figure 5:**
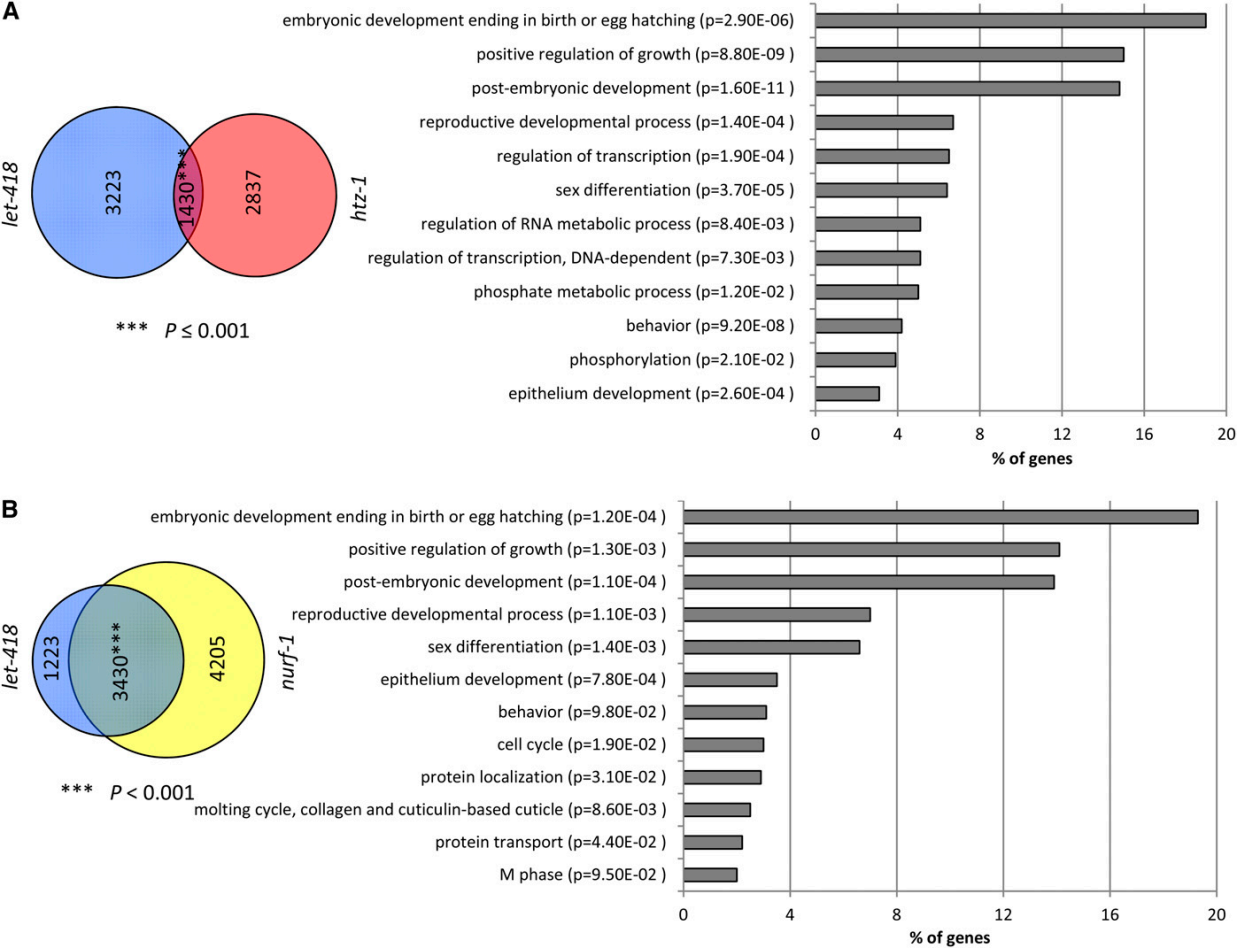
LET-418 and HTZ-1, and LET-418 and NURF-1, share a significant number of common direct targets (*P*-value < 0.001). *P*-values were determined by Fisher’s exact test. Gene ontology categories that are highly enriched for LET-418/HTZ-1 (A) and LET-418/NURF-1 (B) common targets are shown. The percentage of enrichment is indicated by the bars.

In a previous study, we showed that the upregulated genes in *let-418* L1 larvae were enriched in germline-specific genes, including those encoding P granule components (Passannante *et al.* 2010). Since many of our suppressor genes encode chromatin-associated proteins, we tested if they could have an effect on the transcriptional activity of some *let-418* target genes. To approach this question, we selected a subset of germline genes that were found in our list of upregulated genes and measured their transcriptional activity in *let-418*, *nurf-1*, *htz-1*, *let-418*;*nurf-1*, and *let-418*;*htz-1* backgrounds (Passannante *et al.* 2010). As expected, *deps-1*, *pgl-1* (encoding P granule components), and *pie-1* (encoding the germline determinant PIE-1) mRNA levels were increased in *let-418* larvae (Figure 4). Their transcriptional inductions were significantly lowered when *htz-1* or *nurf-1* were depleted by RNAi (Figure 4A), indicating that *nurf-1* and *htz-1* are required for the upregulation of these three genes in *let-418* mutants. Furthermore, we took advantage of available ChIP-seq data for LET-418, NURF-1, and HTZ-1 that were generated by the modENCODE consortium using L3 larvae as starting material (Gerstein *et al.* 2010). We compared their list of target genes to identify common direct targets. The analysis showed that LET-418 and HTZ-1, as well as LET-418 and NURF-1, were sharing a significant number of common target genes in L3 larvae (*P* < 0.001) (Figure 5). GO analysis of the common targets revealed that they are enriched in genes with predicted function in embryonic and postembryonic development, as well as reproductive developmental processes (Huang *et al.* 2009a,b). Unfortunately, the germline genes *deps-1*, *pgl-1*, and *pie-1* that we tested for their transcriptional activity in *let-418*, *nurf-1*, and *htz-1* backgrounds do not figure in this list. Either they are not direct targets or they are not bound by LET-418, NURF-1, and HTZ-1 at the L3 stage, or both, suggesting a dynamic binding of targets during development. These results indicate that the chromatin proteins LET-418, HTZ-1, and NURF-1 are likely recruited to common target genes, consistent with the idea that these chromatin factors are together regulating important developmental processes.

Starved L1 larvae and arrested *let-418* mutants look superficially similar. However, blast cell quiescence in starved larvae, but not in *let-418* animals, depends on DAF-16 (Baugh and Sternberg 2006). Nevertheless, absence of *daf-16* seems to prevent the occurrence of some other defects in the *let-418* mutant larvae, as indicated by the loss of the M cell marker expression (Figure S1). This suggests that DAF-16 is involved in both types of developmental arrest, one due to starvation and the other due to an absence of *let-418* activity. Therefore, we were interested to know if LET-418 and DAF-16 could share common regulated genes. Indeed, a comparison between the transcription profiles of *daf-16* and *let-418* mutants revealed a significant number of commonly regulated targets that, following GO analysis, belong to gene categories involved in metabolic pathways (Figure S6, A and B) (Passannante *et al.* 2010; Kaplan *et al.* 2015). We selected three of these common genes, namely *dct-3*, *W08A12.4*, and *fbxa-165* (Figure S7), and tested if they are also coregulated by a subset of our suppressors. We compared the transcriptional activities of these three genes in *let-418*, *nurf-1*, *htz-1*, or *set-26* animals with that of *let-418*;*htz-1*, *let-418*;*nurf-1*, or *let-418*;*set-26* animals. The mRNA levels of *dct-3*, *W08A12.4*, and *fbxa-165* were increased in *let-418* mutants, and this induction was dependent on *nurf-1*, *htz-1*, and *set-26* activity (Figure 4B). This indicates that LET-418, together with other chromatin-associated factors, is controlling the transcription of a subset of DAF-16 targets that may be involved in larval development.

## Discussion

In the absence of *let-418* maternal gene activity, *let-418* mutants do not initiate postembryonic development. The PGCs and blast cells do not divide. Overall, arrested *let-418* worms resemble L1 diapaused larvae, except that they do not survive and recover as well as starved larvae that have been returned to food. By performing a genome-wide RNAi suppressor screen, we could show that *let-418* developmental arrest is dependent mainly on genes encoding chromatin factors and on a few other genes exhibiting various functions. All the suppressors identified in this study, except the histone methyltransferase-encoding gene *set-26*, are able to suppress not only the developmental defects but also the somatic expression of germline-specific genes associated with the lack of *let-418* activity. In contrast, *set-26* suppresses developmental defects but not the somatic expression of germline genes.

**Figure 6:**
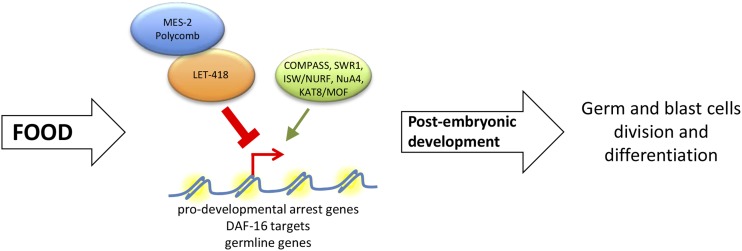
Model for the action of LET-418 and the suppressors. In the presence of food, larval development is initiated and LET-418, together with a network of other chromatin factors, is responsible for modulating the transcription of germline genes, a subset of DAF-16 targets, and some other prodevelopmental arrest genes. This network of chromatin factors includes activator proteins that might compete for regulation at the promoter of target genes and repressors whose localization could be determined by LET-418. MES-2 was shown recently to interact with LET-418 (Käser-Pébernard *et al.* 2016).

In this paper, we describe that a failure of *let-418* worms to initiate postembryonic development was mainly dependent on genes encoding chromatin factors that are members of multi-protein complexes known to activate transcription. Recently, we found that LET-418, together with the histone demethylase SPR-5, blocks the activity of the COMPASS complex to ensure germ cell fate maintenance (Käser-Pébernard *et al.* 2014). A similar mechanism could function at the transition between embryonic and postembryonic development in the absence of food. In such a model (Figure 6), LET-418 could control the access of activator complexes, such as COMPASS, but also ISW/NURF, SWR1, KAT8/MOF, and NuA4, to their target genes, thereby maintaining the quiescence and pluripotency of blast cells. Consistent with this hypothesis, we observed that upregulation of some known LET-418-regulated genes, such as the germline genes *pie-1*, *deps-1*, and *pgl-1*, and a subset of DAF-16 targets, is dependent on NURF-1 or HTZ-1 (Figure 4) (Passannante *et al.* 2010). Altogether, our data suggest that LET-418 could organize the chromatin structure at target genes, the regulation of which is crucial for developmental transitions.

In mammalian cells, Mi2 together with other NuRD components restricts the activity of genes to allow the transition of ES cells from the pluripotent state to lineage commitment (Reynolds *et al.* 2012, 2013). It is proposed that this regulation is necessary to keep cells responsive to developmental cues. In worms, postembryonic development consists essentially of PGC and blast cell division and differentiation. Similar to Mi2 in ES cells, LET-418 could be responsible for the restriction of gene expression and to ensure responsiveness to developmental cues such as the presence of food.

Among our suppressors, we also found genes encoding members of the Polycomb complex, which, like LET-418, is known to repress transcription during development. One possible way to interpret this result is that the suppression effect may occur through MES-4; Polycomb proteins are required for the proper localization of MES-4 exclusively to the autosomes (Fong *et al.* 2002; Bender *et al.* 2004), and depletion of the polycomb proteins MES-2/3/6 could lead to a chromosome-wide relocalization of MES-4, resulting in decreased MES-4 activity at LET-418 target genes (Boyer *et al.* 2006; Lee *et al.* 2006; Yuzyuk *et al.* 2009). An alternative way to interpret the suppressor activity of *mes-2/3/6* is that LET-418 plays a role in the recruitment or localization of Polycomb proteins (Figure 6). This is supported by our recent findings that LET-418 interacts with MES-2 and can recruit it to the chromatin (Käser-Pébernard *et al.* 2016). Absence of *let-418* activity could lead to mistargeting of MES-2 and the repression of genes that are not normally bound by the Polycomb complex, thereby resulting in a developmental arrest of *let-418* animals. Depletion of PRC2 components may remove this repression and allow development in a *let-418* background. This model is also supported by the finding that, in ES cells, Polycomb repressive complex 2 is recruited by NuRD to specific target genes to direct their repression (Reynolds *et al.* 2011).

*set-26* was the only suppressor gene that did not suppress the somatic expression of germline genes in *let-418* larvae. *set-26* is expressed in most, if not all, somatic cells (Ni *et al.* 2011) and the *set-26* gene product methylates lysine 9 of histone H3 *in vitro* (Greer *et al.* 2014), which is usually associated with chromatin compaction and gene repression (Li *et al.* 2007; Bannister and Kouzarides 2011). One possible interpretation is that perturbation of the H3K9 methylation level and localization of this modification could affect chromatin structure, thereby leading to the reactivation of crucial prodevelopmental genes in *let-418*;*set-26* animals. However, such a putative perturbation of the H3K9 methylation pattern would not repress the germline transcription program in somatic cells.

Only five suppressors were identified that did not belong to chromatin factors. As part of the deubiquitylation machinery, MATH-33 might alter the metabolism of protein turnover to an extent that is sufficient to partially suppress the developmental defects of *let-418* (McCloskey and Kemphues 2012). Genes involved in protein metabolism have been found to suppress defects associated with mutations in another transcriptional repressor, *lin-35* (Polley and Fay 2012). This suggests that altering the amounts of some key proteins in the process might be sufficient to trigger the onset of postembryonic development.

Altogether, our suppressor screen uncovered a network of chromatin factors that regulate the transition from embryonic to postembryonic development. This transition involves crucial decisions, such as exit from the cell cycle of the blast cells, cell proliferation, and differentiation. Strict control of these processes is critical during development, but also to prevent aberrant proliferation and differentiation during tumorigenesis. Analyzing how these various chromatin factors, together with LET-418, are recruited to key developmental genes will contribute to our understanding of the functional importance of chromatin in the response to developmental cues.

## Supplementary Material

Supplemental material is available online at www.g3journal.org/lookup/suppl/doi:10.1534/g3.116.037747/-/DC1.
