# Supplementary material for: A Network of Chromatin Factors Is Regulating the Transition to Postembryonic Development in Caenorhabditis elegans
Source: G3 (Bethesda). 2016 Dec 22;7(2):343–53. doi: 10.1534/g3.116.037747 (PMC5295584; doi:10.1534/g3.116.037747)
Supplement: Supplementary file 1 [file 343FigureS1.pptx]

## Slide 1
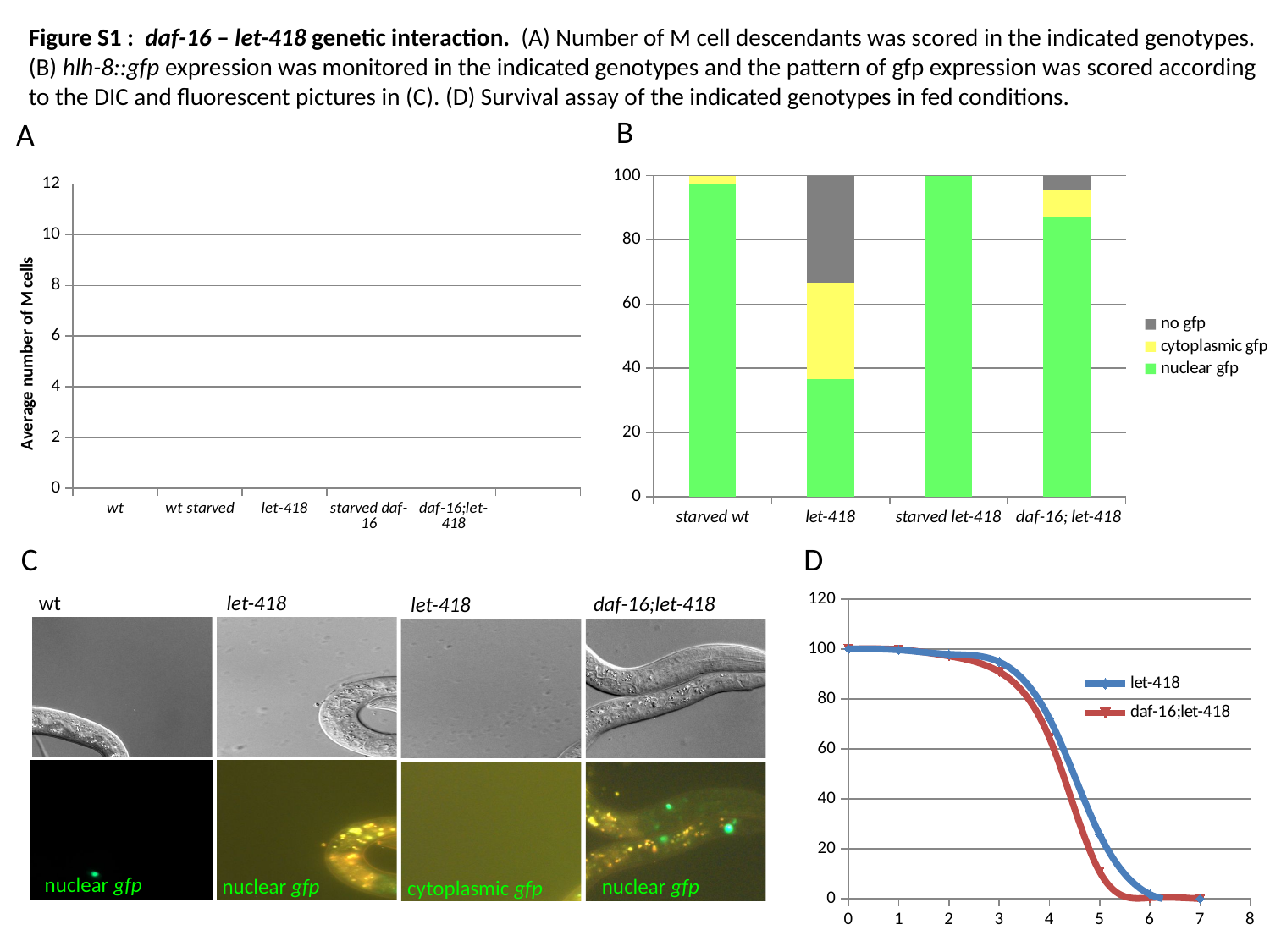

Figure S1 : daf-16 – let-418 genetic interaction. (A) Number of M cell descendants was scored in the indicated genotypes. (B) hlh-8::gfp expression was monitored in the indicated genotypes and the pattern of gfp expression was scored according to the DIC and fluorescent pictures in (C). (D) Survival assay of the indicated genotypes in fed conditions.
### Chart
| Category | |
|---|---|
| wt | 14.6 |
| wt starved | 1.0 |
| let-418 | 0.666666666666667 |
| starved daf-16 | 1.91780821917808 |
| daf-16;let-418 | 0.957142857142857 |B
A
### Chart
| Category | nuclear gfp | cytoplasmic gfp | no gfp |
|---|---|---|---|
| starved wt | 97.61904761904762 | 2.380952380952381 | 0.0 |
| let-418 | 36.66666666666638 | 30.0 | 33.33333333333333 |
| starved let-418 | 100.0 | 0.0 | 0.0 |
| daf-16; let-418 | 87.14285714285673 | 8.571428571428571 | 4.285714285714285 |C
D
### Chart
| Category | let-418 | daf-16;let-418 |
|---|---|---|let-418
wt
daf-16;let-418
let-418
nuclear gfp
nuclear gfp
nuclear gfp
cytoplasmic gfp
