# Supplementary material for: A Network of Chromatin Factors Is Regulating the Transition to Postembryonic Development in Caenorhabditis elegans
Source: G3 (Bethesda). 2016 Dec 22;7(2):343–53. doi: 10.1534/g3.116.037747 (PMC5295584; doi:10.1534/g3.116.037747)
Supplement: Supplementary file 2 [file 343FigureS2.pptx]

## Slide 1
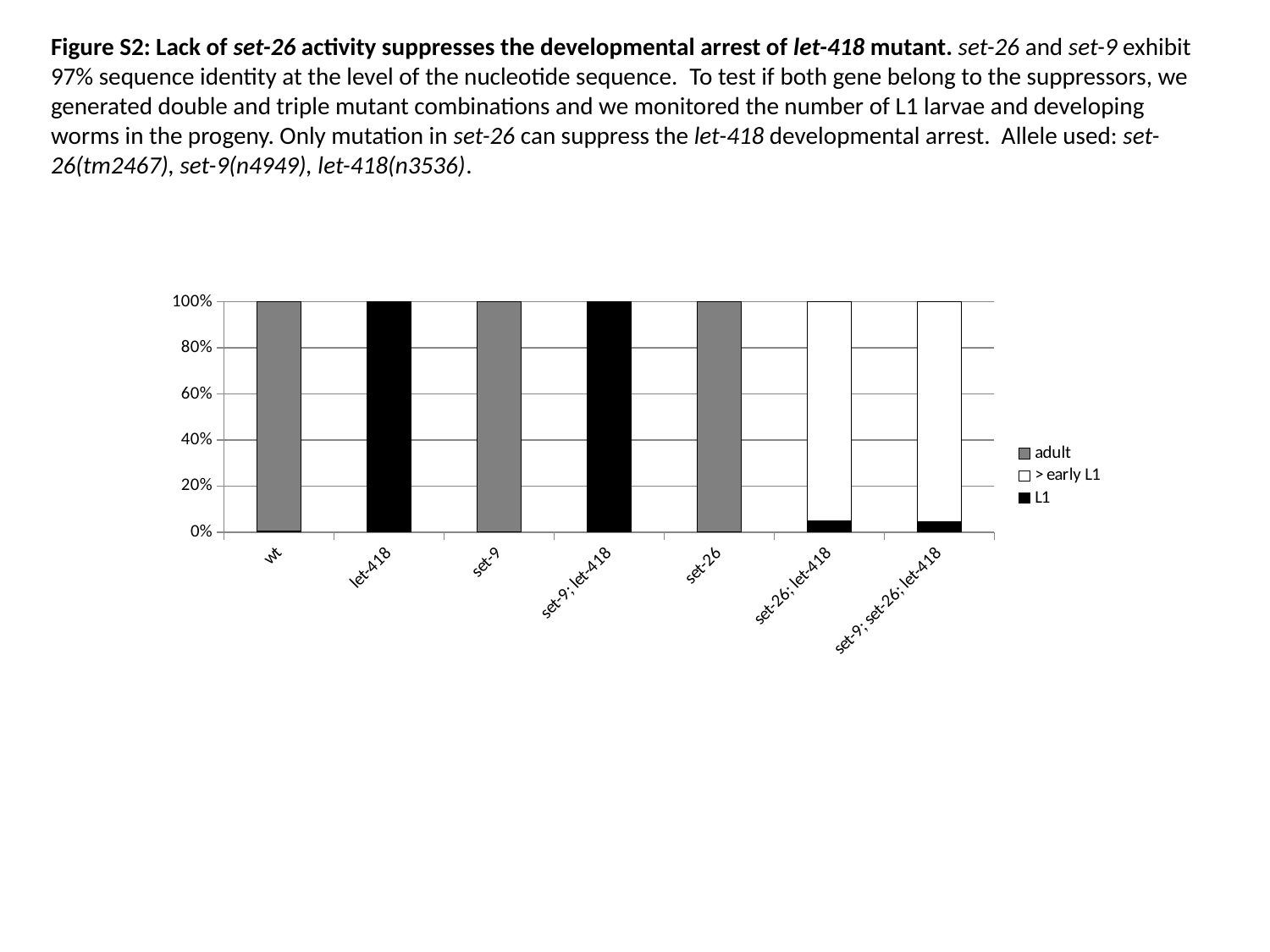

Figure S2: Lack of set-26 activity suppresses the developmental arrest of let-418 mutant. set-26 and set-9 exhibit 97% sequence identity at the level of the nucleotide sequence. To test if both gene belong to the suppressors, we generated double and triple mutant combinations and we monitored the number of L1 larvae and developing worms in the progeny. Only mutation in set-26 can suppress the let-418 developmental arrest. Allele used: set-26(tm2467), set-9(n4949), let-418(n3536).
### Chart
| Category | L1 | > early L1 | adult |
|---|---|---|---|
| wt | 1.0 | 0.0 | 309.0 |
| let-418 | 228.0 | 0.0 | 0.0 |
| set-9 | 0.0 | 0.0 | 233.0 |
| set-9; let-418 | 159.0 | 0.0 | 0.0 |
| set-26 | 0.0 | 0.0 | 100.0 |
| set-26; let-418 | 1.0 | 19.0 | 0.0 |
| set-9; set-26; let-418 | 3.0 | 62.0 | 0.0 |
