# Supplementary material for: A Network of Chromatin Factors Is Regulating the Transition to Postembryonic Development in Caenorhabditis elegans
Source: G3 (Bethesda). 2016 Dec 22;7(2):343–53. doi: 10.1534/g3.116.037747 (PMC5295584; doi:10.1534/g3.116.037747)
Supplement: Supplementary file 4 [file 343FigureS4.pptx]

## Slide 1
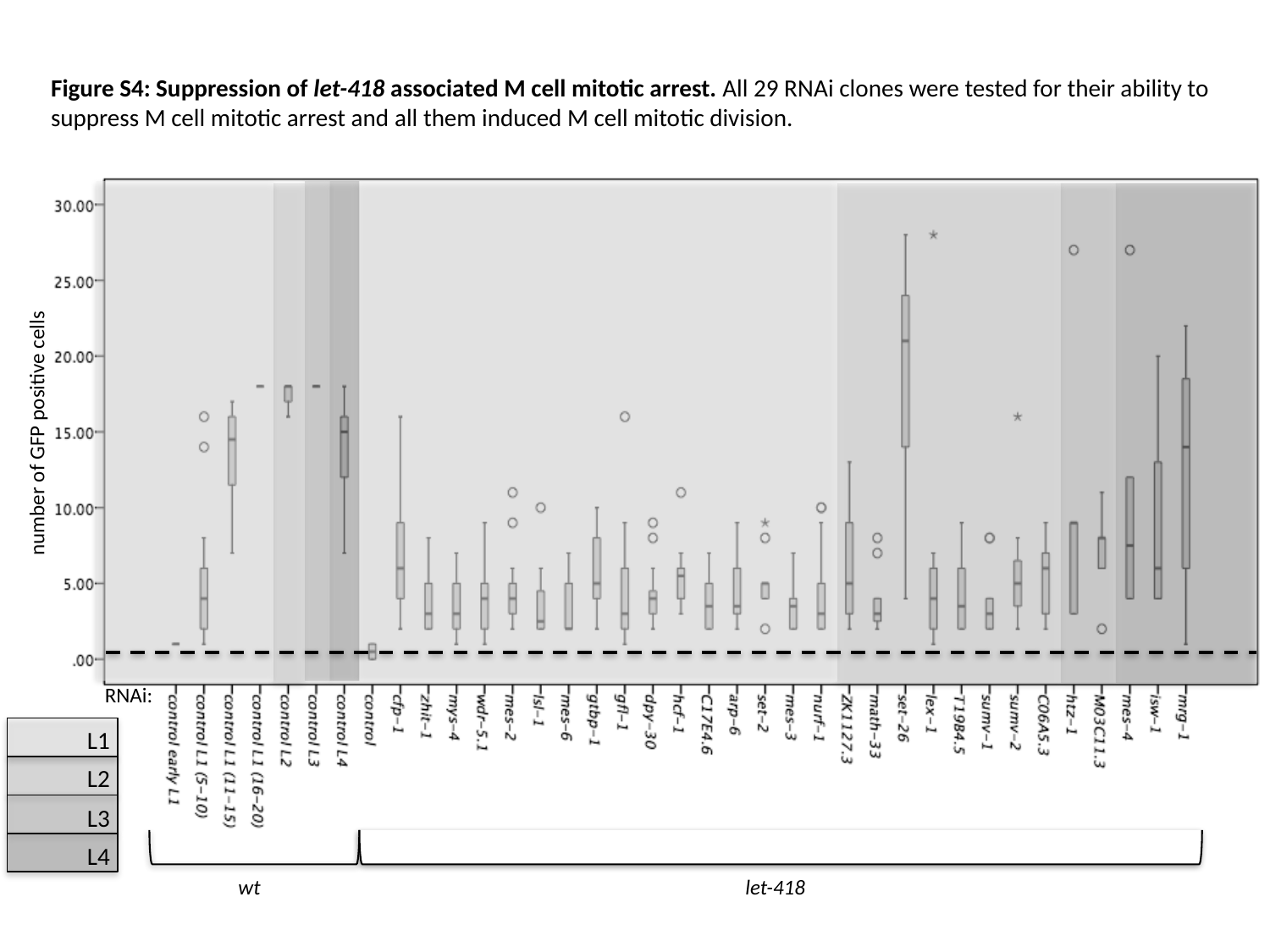

Figure S4: Suppression of let-418 associated M cell mitotic arrest. All 29 RNAi clones were tested for their ability to suppress M cell mitotic arrest and all them induced M cell mitotic division.
number of GFP positive cells
RNAi:
L1
L2
L3
L4
wt
let-418
