# Supplementary material for: A Network of Chromatin Factors Is Regulating the Transition to Postembryonic Development in Caenorhabditis elegans
Source: G3 (Bethesda). 2016 Dec 22;7(2):343–53. doi: 10.1534/g3.116.037747 (PMC5295584; doi:10.1534/g3.116.037747)
Supplement: Supplementary file 6 [file 343FigureS6.pptx]

## Slide 1
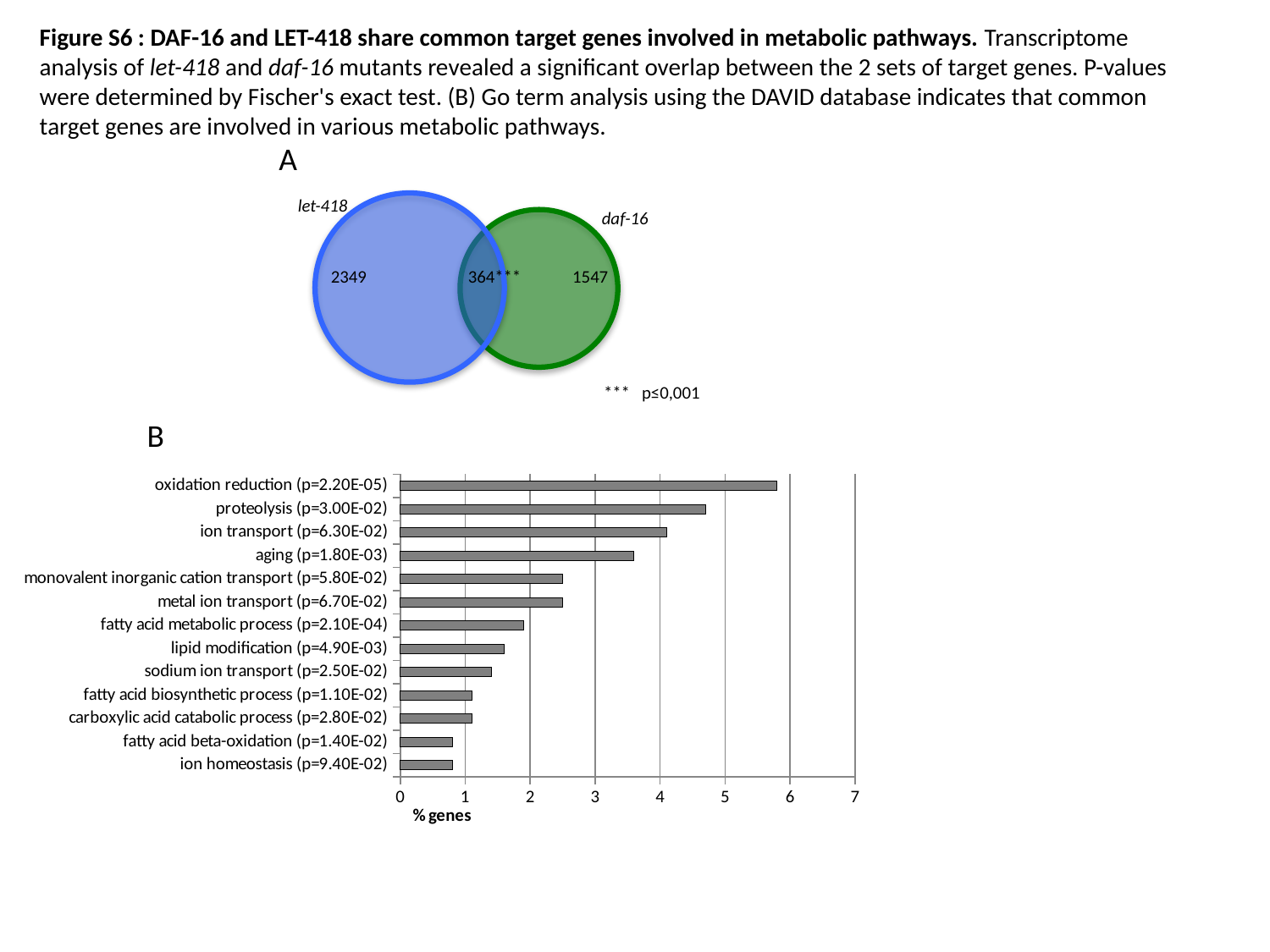

Figure S6 : DAF-16 and LET-418 share common target genes involved in metabolic pathways. Transcriptome analysis of let-418 and daf-16 mutants revealed a significant overlap between the 2 sets of target genes. P-values were determined by Fischer's exact test. (B) Go term analysis using the DAVID database indicates that common target genes are involved in various metabolic pathways.
A
let-418
daf-16
2349
364***
1547
*** p≤0,001
B
### Chart
| Category | |
|---|---|
| ion homeostasis (p=9.40E-02) | 0.8 |
| fatty acid beta-oxidation (p=1.40E-02) | 0.8 |
| carboxylic acid catabolic process (p=2.80E-02) | 1.1 |
| fatty acid biosynthetic process (p=1.10E-02) | 1.1 |
| sodium ion transport (p=2.50E-02) | 1.4 |
| lipid modification (p=4.90E-03) | 1.6 |
| fatty acid metabolic process (p=2.10E-04) | 1.9 |
| metal ion transport (p=6.70E-02) | 2.5 |
| monovalent inorganic cation transport (p=5.80E-02) | 2.5 |
| aging (p=1.80E-03) | 3.6 |
| ion transport (p=6.30E-02) | 4.1 |
| proteolysis (p=3.00E-02) | 4.7 |
| oxidation reduction (p=2.20E-05) | 5.8 |
