# Supplementary material for: A Network of Chromatin Factors Is Regulating the Transition to Postembryonic Development in Caenorhabditis elegans
Source: G3 (Bethesda). 2016 Dec 22;7(2):343–53. doi: 10.1534/g3.116.037747 (PMC5295584; doi:10.1534/g3.116.037747)
Supplement: Supplementary file 7 [file 343FigureS7.pptx]

## Slide 1
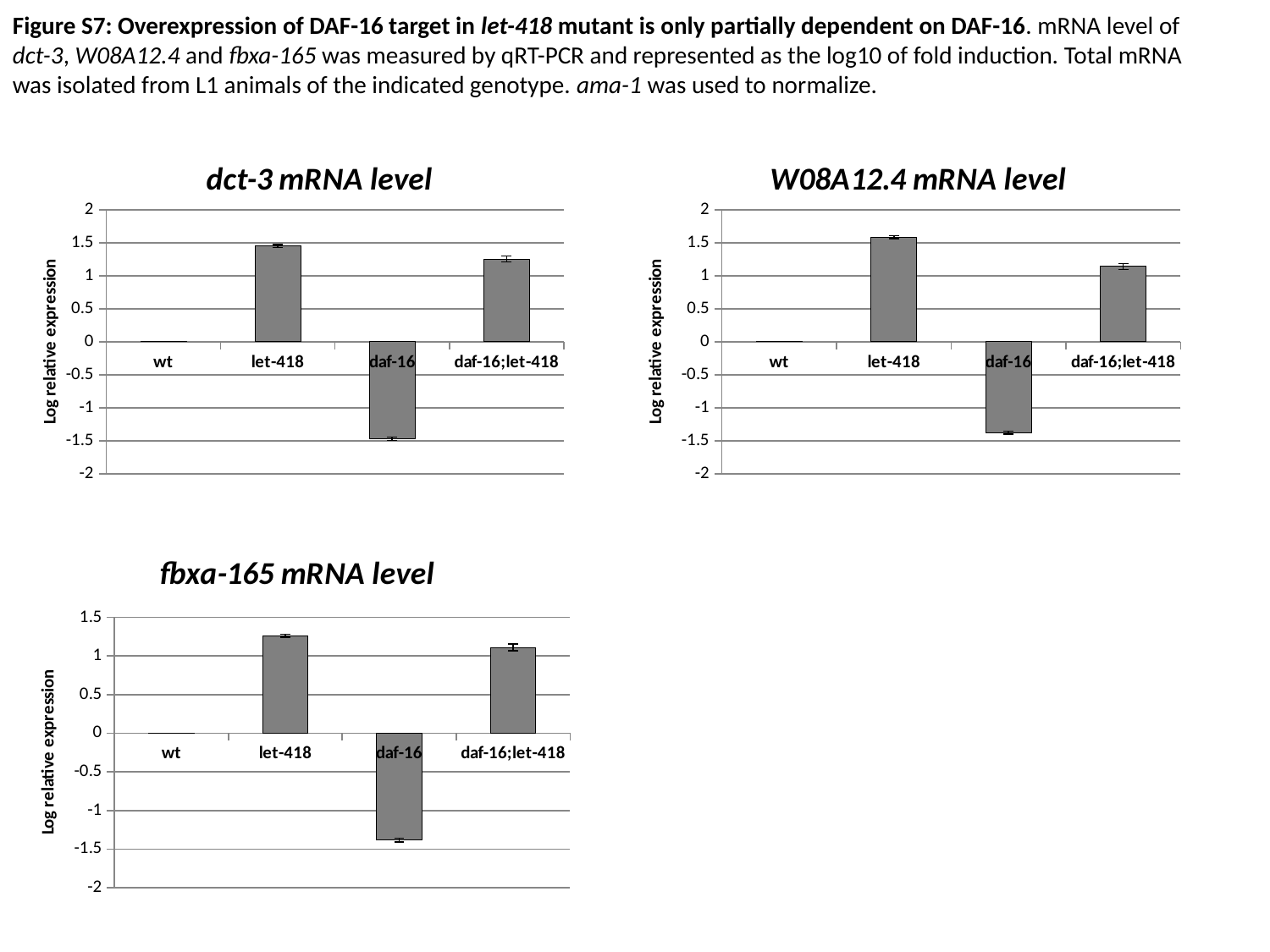

Figure S7: Overexpression of DAF-16 target in let-418 mutant is only partially dependent on DAF-16. mRNA level of dct-3, W08A12.4 and fbxa-165 was measured by qRT-PCR and represented as the log10 of fold induction. Total mRNA was isolated from L1 animals of the indicated genotype. ama-1 was used to normalize.
### Chart: dct-3 mRNA level
| Category | |
|---|---|
| wt | 0.0 |
| let-418 | 1.450249108319361 |
| daf-16 | -1.467594290404534 |
| daf-16;let-418 | 1.252853030979893 |
### Chart: W08A12.4 mRNA level
| Category | |
|---|---|
| wt | 0.0 |
| let-418 | 1.584331224367531 |
| daf-16 | -1.375541722295432 |
| daf-16;let-418 | 1.139879086401236 |
### Chart: fbxa-165 mRNA level
| Category | |
|---|---|
| wt | 0.0 |
| let-418 | 1.260224502766158 |
| daf-16 | -1.382504337285879 |
| daf-16;let-418 | 1.11058971029925 |
